# Clinical value for the detection of fetal chromosomal deletions/duplications by noninvasive prenatal testing in clinical practice

**DOI:** 10.1002/mgg3.1687

**Published:** 2021-05-05

**Authors:** Lingshan Gou, Feng Suo, Yi Wang, Na Wang, Qin Wu, Shunan Hu, Peng Wang, Lize Gu, Man Zhang, Chuanxia Wang, Yan Zhang, Xin Yin, Peng Zhang, Jian Xu, Xingqi Wang, Maosheng Gu

**Affiliations:** ^1^ Center for Genetic Medicine Xuzhou Maternity and Child Health Care Hospital Affiliated to Xuzhou Medical University Xuzhou China; ^2^ Department of Technology Suzhou Beikang Medical Device Co. Ltd. Suzhou China; ^3^ Zhejiang Biosan Biochemical Technologies Co. Ltd. Hangzhou China; ^4^ Office of Scientific Research & Henan Provincial Key Laboratory of Children’s Genetics and Metabolic Diseases Children’s Hospital Affiliated to Zhengzhou University Zhengzhou China; ^5^ Shenzhen Longgang Institute of Stomatology Shenzhen Longgang E.N.T. Hospital Shenzhen China; ^6^ Key Laboratory for Biotechnology on Medicinal Plants of Jiangsu Province School of Life Science Jiangsu Normal University Xuzhou China

**Keywords:** chromosomal microarray analysis, deletion, duplication, fetus, NIPT, prenatal diagnosis

## Abstract

**Objective:**

This study was to report the experiences on the clinical value of noninvasive prenatal testing (NIPT) for the screening of fetal chromosomal deletions/duplications.

**Methods:**

We performed a retrospective analysis of a cohort of 20,439 pregnancies undergoing NIPT from March 2017 to September 2020 at a single center. Patients with positive NIPT results for fetal chromosomal deletions or duplications had options of invasive diagnostic testing or no further testing. The data were complied from all cases with positive NIPT results for chromosomal deletions/duplications. The positive predictive value (PPV) was calculated from tabulated data.

**Results:**

In this cohort, positive NIPT results for fetal chromosomal deletions/duplications were found in 60 pregnant women. Of the positive samples, further invasive testing was performed in 39 cases, in which 9 cases were found to be true positive. The overall PPV for chromosomal deletions/duplications was 23.1%. In addition, fetal structural anomaly was found by ultrasound examination in three cases, in which the chromosomal deletions/duplications of three cases were not verified. Moreover, an unexpected pathogenic 8p23.3 deletion was identified by invasive testing in 1 fetus with a false positive NIPT screen for 3q27.3q29 duplication.

**Conclusions:**

In summary, positive NIPT results of chromosomal deletions/duplications were not uncommon in clinical practice, whereas the PPV for the testing was low. Hence, potential risks and high percentage of false positives for these abnormal NIPT results might be informed to pregnant women before the choice made of invasive testing.

## Graphical abstract

Confirmatory invasive diagnostic testing might be used for the positive noninvasive prenatal testing (NIPT) screens of fetal chromosomal deletions/duplications in clinical practice, while caution for the low accuracy should be noted. Moreover, maternal chromosomal aberrations were important factors affecting the accuracy of NIPT for the detection of fetal chromosomal deletions/duplications.

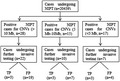

## 1 INTRODUCTION

Noninvasive prenatal testing (NIPT) is a technology detecting fetal aneuploidy through genome‐wide sequencing of cell‐free fetal DNA (cffDNA) from maternal circulation. In recent years, NIPT has been widely used as a primary or second‐tier screening test for fetal common chromosomal aneuploidy (trisomy 13, 18, or 21) detection due to the higher sensitivity and lower false positive rate than maternal serum screening test (Porreco et al., 2014; Song et al., 2015). Moreover, large evidence also demonstrates that NIPT has good performance in fetal sex chromosomal aneuploidy detection in clinical practice (Kornman et al., 2017; Song et al., 2015; Suo et al., 2018).

Copy number variants (CNVs) arising from the chromosomal deletions/duplications are associated with chromosome syndromes and genetic disorders (Carvalho & Lupski, 2016; Stankiewicz & Lupski, 2010). Previous reports demonstrate that clinically relevant deletions/duplications occur in 1.7% of structurally normal pregnancies, and also in 6.0% of fetuses with structural abnormalities (Levy & Wapner, 2018). Identification of the CNVs is benefit to genetic analysis of fetus with congenital anomalies (Wapner et al., 2012). Thus, there is an urgent clinical need in prenatal screening for chromosomal deletions/duplications as the additional test for the screening of common autosomal aneuploidy. Several studies have reported the clinical utility of NIPT in the screening of fetal chromosomal deletions/duplications (Helgeson et al., 2015; Hu et al., 2019; Li et al., 2016; Martin et al., 2018; Schwartz et al., 2018; Zheng et al., 2019). However, the cited studies do not represent the variable NIPT platforms and screening algorithms currently used in clinical practice. More clinical information is required on the application of this technique.

In clinical practice, chromosomal deletions/duplications are often reported as the additional findings of NIPT report. However, more evidence on the accuracy of NIPT in the screening of chromosomal deletions/duplications is needed to facilitate genetic counseling. Before opting for invasive diagnostic procedure, the risk and expenditure of such procedures as well as the potential risk of chromosomal deletions/duplications should be fully considered. In the current study, we reviewed the screening results of chromosomal deletions/duplications detected by NIPT and the follow‐up diagnostic testing over the past 3.5 years at a single center.

## 2 MATERIALS AND METHODS

### 2.1 Patients

In the present study, we performed a retrospective analysis of a cohort of 20,439 pregnancies undergoing NIPT from March 2017 to September 2020 at the Center for Genetic Medicine of Maternity and Child Health Care Hospital affiliated to Xuzhou Medical University. Informed written consent was obtained from all patients, and they all received the pretest counseling for NIPT. Peripheral blood sample of 5 ml from each pregnant woman (12–23 weeks’ gestation) was collected into ethylenediaminetetraacetic acid (EDTA) tube and stored immediately after sampling.

### 2.2 Maternal cffDNA sequencing

After a two‐step centrifugation process, cffDNA was extracted from plasma using a nucleic acid isolation kit according to the manufacturer's instructions (Darui Biotechonology Co., Ltd., Guangzhou, China). Following library construction and quality control, the libraries were pooled and sequenced on an Ion Proton semiconductor sequencer (Life Technologies, Carlsbad, CA, USA). Briefly, the isolated DNA was end‐repaired with T4 DNA polymerase and T4 polynucleotide kinase, then ligated to barcode adapter using DNA ligase. After amplification by polymerase chain reaction (PCR), libraries were subject to double‐size selection for removing residual adaptors and primers with Agencourt AMPure XP beads (Darui Biotechnology Co., Ltd., Guangzhou, China). Following quantification with the Ion Library Quantitation Kit (Thermo Fisher, Eugene, OR, USA), the libraries were loaded onto an Ion semiconductor chip for sequencing. Combined GC base correction and *Z*‐score predication were used to determine fetal CNVs. The chromosomal region with *Z*‐score >3 or < −3 was classified as positive of fetal chromosomal deletions/duplications. The clinical significantly CNVs larger than 500 kb detected by NIPT were reported before confirmation was recommended. Moreover, the reported CNVs in this study included recurrent and rare chromosomal deletions/duplications.

### 2.3 Confirmatory invasive testing

Amniocentesis, G‐band karyotype analysis, and chromosome microarray analysis (CMA) were all provided to each pregnancy opting for invasive testing due to the positive NIPT results. G‐banding karyotype of cultured amniotic fluid cells was performed as described previously (Porreco et al., 2014). For the test of CMA, genomic DNA was extracted from uncultured fresh amniotic fluid cells using QIAamp DNA Blood Mini Kit (Qiagen Inc., Germany). Subsequently, the DNA underwent sequential processes including digestion, PCR, PCR‐product check, purification, quantification, fragmentation, QC gel labeling, hybridization, washing, and staining, following by the scanning on an Affymetrix GeneChip Scanner. The data were visualized and then analyzed using Affymetrix Chromosome Analysis Suite (ChAS) Software (Affymetrix, Santa Clara, CA) referring the human assembly GRCh37/hg19. CNVs classification and interpretation were performed in querying Online Mendelian Inheritance in Man (OMIM) database, database of Chromosomal Imbalance and Phenotype in Humans using Ensemble Resources (DECIPHER), database of the Clinical Genome Resource (ClinGen), and the University of California Santa Cruz (UCSC) Genome Browser Database, Database of Genomic Variants, and PubMed. The reporting threshold for CNVs detected by CMA was set at 500 kb with a marker counting more than 50 for gains, and 200 kb with a marker counting more than 50 for losses.

### 2.4 Sequencing of the DNA from maternal circulating leukocyte

The maternal blood samples were centrifuged at 1600× *g* for 10 min at 4℃, followed by collecting 200 μl of peripheral blood leukocytes layer. Genomic DNA was extracted from the blood leukocytes using QIAamp DNA Blood Mini Kit according to the manufacture's instruction. A total of 300 ng genomic DNA was digested with dsDNA fragmentase for 25 min at 37℃. After purification, the digested DNA was end‐repaired with T4 DNA polymerase and T4 polynucleotide kinase, followed by the barcode adapter ligation using DNA ligase. After amplification by PCR, libraries were subject to double‐size selection for removing residual adaptors and primers with Agencourt AMPure XP beads. The libraries were quantified using the Ion Library Quantitation Kit (Thermo Fisher, Eugene, OR, USA). After quality checking, the libraries were loaded onto the Ion semiconductor chip for sequencing. All sequencing data were aligned to the human genome reference sequences (GRCh37/hg19) using TMAP software. Plots of the log of read count were generated for each of the chromosome to track the mean CNVs. The reporting threshold for CNVs was set at 500 kb for gains and losses.

## 3 RESULTS

In the current study, 20,439 pregnant women underwent prenatal screening by NIPT after pretest counseling. Of these samples, positive NIPT results for chromosomal deletions/duplications were found in 60 pregnancies. Advanced maternal age (≥35 years) was the indication for the testing in eight pregnancies with positive NIPT results, and abnormal ultrasound finding was the indication in one positive NIPT case. In addition, 29 positive NIPT cases were found with the indication of abnormal serum biochemical screening results, including high risk and intermediate risk for fetal aneuploidy. Out of the 60 positive cases, diagnostic testing and/or clinical phenotypes were available for 41 pregnancies. Nine of the 39 cases were found to have chromosomal deletions/duplications *via* karyotype and CMA (Table 1). Two cases without invasive diagnostic testing result opted for terminating pregnancy due to the fetal malformation detected by ultrasound examination. Thirty cases were confirmed to be false positive (Tables 1 and S1).

**TABLE 1:** NIPT results for chromosomal deletions/duplications validated by CMA and karyotpye analysis of amniocytes

Sample ID	MA (Y)	GA (W)	Clinical indications	NIPT results	CMA/karyotype analysis results		Maternal sequencing results	Classification/follow up
					Location	Size (Mb)		
*Consistent CNVs detected by NIPT and amniocytes testing*								
Case 1	29	16	Intermediate risk	Dup 1q24.3q25.2 (7.35 Mb)	Dup 1q24.3q25.2	7.56	ND	Pathogenic
Case 2	26	22	NA	Del 4q12q21.23 (27.81 Mb)	Del 4q12q21.1	19.54	Normal	Pathogenic
Case 3	22	23	NA	Del 5p15.33p14.1 (27.30 Mb)	Del 5p15.33p13.3	30.74	Normal	Pathogenic
Case 4	23	24	NA	Del 15q11.2q13.1 (5.69 Mb) (15q11q13 recurrent region)	Del 15q11.2q13.1	5.20	ND	Pathogenic
Case 5	30	19	NA	Dup 17p11.2 (5.01 Mb) (17p11.2 recurrent region)	Dup 17p12p11.2	4.93	Normal	Pathogenic
Case 6	29	19	Intermediate risk	Dup 21q21.1q21.3 (10.08 Mb)	Dup 21q21.1q21.3	10.35	ND	Pathogenic
Case 7	23	18	High risk	Dup 22q11.21 (2.84 Mb) (22q11.2 recurrent region)	Dup 22q11.21	3.27	Dup 22q11.21 (3.28 Mb)	Likely pathogenic
Case 8	19	17	Fetal structural abnormality	Del 22q11.21 (3.20 Mb) (22q11.2 recurrent region)	Del 22q11.21	3.15	Normal	Pathogenic
Case 9	37	19	Advanced maternal age	Del 22q11.21 (1.88 Mb) (22q11.2 recurrent region)	Del 22q11.21	0.99	Del(22)(22q11.21 (0.99 Mb)	Pathogenic
*Inconsistent CNVs detected by NIPT and amniocytes testing*								
Case 10	24	19	Intermediate risk	Del 1p33p31.3 (18.30 Mb)	Normal	–	ND	NA
Case 11	40	19	Advanced maternal age	Dup 1q31.2q32.2 (18.75 Mb); Del 5p15.33p14.1 (26.73 Mb)	Normal	–	ND	NA
Case 12	28	19	NA	Dup 2p25.3p23.3 (25.71 Mb)	Normal	–	ND	NA
Case 13	35	21	Advanced maternal age	Dup 2q32.3q33.2 (8.28 Mb)	Dup 2q31.2	0.52	NA	Uncertain significance
Case 14	28	22	NA	Del 2q33.1q33.3 (8.28 Mb)	Normal	–	ND	Normal liveborn
Case 15	23	18	Intermediate risk	Dup 3q27.3q29 (10.90 Mb) (3q29 recurrent region)	Del 8p23.3	2.01	ND	Pathogenic
Case 16	30	19	Intermediate risk	Dup 4q12q13.2 (13.94 Mb)	Normal	–	ND	NA
Case 17	31	20	NA	Dup 4q31.21q35.2 (46.57 Mb); Dup 16q21q24.3 (24.53 Mb)	Normal	–	ND	NA
Case 18	22	19	NA	Del 4q28.3q31.21 (5.10 Mb)	Normal	–	ND	NA
Case 19	28	18	Intermediate risk	Del 5p15.33p15.1 (16.33 Mb)	Normal	–	ND	Normal liveborn
Case 20	34	20	Intermediate risk	Del 5p15.33p15.2 (10.28 Mb)	Dup Yq11.223	4.23	ND	Uncertain significance
Case 21	33	16	NA	Del 5p15.33p15.1 (16.02 Mb)	Normal	–	ND	Normal liveborn
Case 22	38	16	Advanced maternal age	Del 5p15.33p14.1 (26.17 Mb)	Normal	–	ND	Normal liveborn
Case 23	20	17	Intermediate risk	Del 5p15.33p14.1 (28.83 Mb)	Normal	–	ND	Normal liveborn
Case 24	28	20	High risk	Del 5p15.33p14.3 (23.04 Mb)	Normal	–	ND	NA
Case 25	39	19	NA	Del 5p15.33p15.2 (10.50 Mb; Dup 6p25.3p12.2 (50.32 Mb)	Normal	–	ND	NA
Case 26	25	23	Intermediate risk	Dup 7q31.2q36.3 (41.86 Mb)	Normal	–	ND	Normal liveborn
Case 27	29	18	Intermediate risk	Dup 8q24.13q24.3 (16.58 Mb)	Normal	–	ND	NA
Case 28	28	19	NA	Dup 9p21.3p13.2 (15.26 Mb)	Normal	–	ND	NA
Case 29	32	17	NA	Dup 9q12q21.3 (9.13 Mb)	Normal	–	ND	NA
Case 30	25	22	Intermediate risk	Dup 15q24.3q26.1 (15.38 Mb)	Normal	–	ND	Normal liveborn
Case 31	26	18	High risk	Del 15q11.2q13.1 (5.69 Mb) (15q11q13 recurrent region)	Normal	–	ND	Normal liveborn
Case 32	25	18	NA	Del 15q11.2q13.1 (5.69 Mb) (15q11q13 recurrent region)	Normal	–	Normal	Normal liveborn
Case 33	24	19	NA	Del 15q21.2q23 (18.34 Mb)	Normal	–	ND	NA
Case 34	36	17	Advanced maternal age	Dup 16p13.12p12.3 (5.66 Mb)	Normal	–	ND	Normal liveborn
Case 35	31	21	NA	Del 16q23.1q24.2 (11.13 Mb)	Normal	–	ND	NA
Case 36	37	17	Advanced maternal age	Dup 18p11.32p11.31 (3.12 Mb)	Normal	–	ND	NA
Case 37	35	19	Advanced maternal age	Dup 22q11.21 (2.94 Mb) (22q11.2 recurrent region)	Normal	–	Dup 22q11.21 (2.92 Mb)	Normal liveborn
Case 38	34	19	NA	Del 22q11.21q11.3 (4.71 Mb) (22q11.2 recurrent region)	Normal	–	Normal	Normal liveborn
Case 39	26	18	NA	Del 22q11.21q11.22 (4.71 Mb) (22q11.2 recurrent region)	Normal	–	ND	NA

Abbreviations: CMA, chromosomal microarray analysis; CNVs, copy number variants; Del, deletion; Dup, duplication; GA, gestational age; MA, maternal age; NA, not available; ND, not detected; NIPT, noninvasive prenatal testing; W, week; Y, year.

In these cases, positive NIPT results for 22q11.2 duplication were noted in seven cases, in which two cases underwent invasive testing. Of these two cases, one case was false positive caused by maternal CNV, and a maternal inherited 22q11.2 duplication was observed in the true positive case. The positive predictive value (PPV) for 22q11.2 duplication detection by NIPT was 50.0%. In addition, positive results of 22q11.2 deletion were reported in six cases at NIPT. Of these samples, two cases declined further invasive testing; two cases were false positive and the other two cases were true positive (PPV = 50.0%). Of the two true positive cases, the CNV was de novo in one case, whereas the CNV in the other one was maternally inherited. Positive NIPT results for 1q24.3q25.2 duplication, 4q12q21.1 deletion, 17p12p11.2 duplication, and 21q21.1q21.3 duplication were observed in 1 pregnancy, respectively. The values of PPV for these four CNVs types were all 100.0%. Moreover, a total of four patients were found by NIPT to have a 5p15.33p13.3 deletion with one case confirmed to be true positive (PPV = 25.0%). At NIPT, 15q11.2q13.1 deletion was found in four cases, in which three cases underwent further invasive testing. Consequently, one positive NIPT case with 15q11.2q13.1 deletion was confirmed by invasive diagnostic testing (33.3%). In addition, one case of suspected 12p13.33p11.1 duplication and one case of suspected 22q11.21q12.1 duplication at NIPT chose pregnancy termination due to abnormal ultrasound findings. Moreover, an unexpected pathogenic 8p23.3 deletion was found via CMA in one pregnancy with a false positive NIPT result for 3q27.3q29 duplication (Tables 1 and S1).

In these positive NIPT cases, 28 CNVs were larger than 10 Mb, 15 CNVs were between 5 and 10 Mb, and 17 CNVs were smaller than 5 Mb in size. For the 28 positive NIPT samples with CNVs > 10 Mb, invasive testing were performed in 22 cases, including 3 true positive and 19 false positive cases, respectively. As for the 17 CNVs between 5 and 10 Mb at NIPT, 10 cases received further invasive testing. Of these 10 cases, three pregnancies were true positive, whereas 7 cases were false positive. As for the 17 CNVs < 5 Mb reported by NIPT, 7 cases underwent further invasive testing. Invasive diagnosis confirmed the true positive CNVs in three pregnancies, and the false positive CNVs in four cases. The overall PPV for chromosomal deletions/duplications detection by NIPT was 23.1%. Moreover, the PPV for detecting chromosomal deletions/duplications at size of >10 Mb, between 5 and 10 Mb, and smaller than 5 Mb were 13.6%, 30.0%, and 42.9%, respectively (Table 2).

**TABLE 2:** Evaluation of the positive predictive value (PPV) of NIPT in detecting chromosomal deletions/duplications

CNVs size	Positive cases	Cases undergoing amniocytes testing	TP cases	FP cases	PPV (%)
>10 Mb	28	22	3	19	13.6
5−10 Mb	15	10	3	7	30.0
<5 Mb	17	7	3	4	42.9
Total	60	39	9	30	23.1

Abbreviations: CNVs, copy number variants; FP, false positive; PPV, positive predictive value; TP, true positive.

## 4 DISCUSSION

During recent years, high‐throughout sequencing of fetal cffDNA in maternal circulation has undergone rapid expansion on both utilization and coverage. However, the clinical utilization of this technology in detecting fetal chromosomal deletions/duplications remains controversial. A published case report shows that NIPT detects a fetal 21q11.2q22.11 deletion (~18 Mb), which is verified by testing of CMA (Zheng et al., 2019). In addition, several other cases with fetal chromosomal deletions/duplications detected by NIPT have been reported recently (Yin et al., 2019; Zhao et al., 2019). Furthermore, several investigators report their clinical experiences on the detection of chromosomal deletions/duplications using NIPT (Helgeson et al., 2015; Hu et al., 2019; Li et al., 2016; Martin et al., 2018; Schwartz et al., 2018; Zheng et al., 2019). However, the PPV for chromosomal deletions/duplications in these studies varies largely. More clinical evidence regarding the clinical utility of this screening tool for the detection of chromosomal deletions/duplications is required to educate the clinicians and patients.

In the current study, we reviewed the clinical experiences to noninvasively detect deletions/duplications at a single center. Among a total cohort of 20,439 pregnant women undergoing NIPT for the screening of trisomy 13, 18, or 21, the positive results of clinically significant CNVs were reported as Supporting Information in 60 pregnancies. Karyotype analysis and CMA were performed in 39 positive NIPT cases, finding 9 true positive cases. The PPV in this tested population is 23.1%, which was similar with previous study (Martin et al., 2018). In another published report, the PPV for chromosomal deletions screened by NIPT is 8.9%, which is lower than our results (Schwartz et al., 2018). However, the PPV in our cohort was lower than that of some other studies (Helgeson et al., 2015; Hu et al., 2019; Li et al., 2016).

Multiple reasons including fetal fraction, total number of DNA fragments counts, fetal and/or placental mosaicism, maternal chromosome aberration, co‐twin demise, and even laboratory error are recognized as factors affecting the test performance of NIPT for the screening of fetal aneuploidies (Canick et al., 2013; Choi et al., 2013; Porreco et al., 2014; Wang et al., 2014). Though the performances vary largely in previous studies, the factors involving in the discordant NIPT results of chromosomal deletions/duplications remained obscured. Fetal DNA fraction, the size of the chromosomal deletions/duplications as well as the sequencing coverage and depth have been reported to affect the accuracy for chromosomal deletions/duplications detection (Petersen et al., 2017; Zhang et al., 2019; Zhao et al., 2015). In this study, we found that one false positive result of 22q11.2 duplication was due to the maternal copy number variant. Thus, although NIPT is designed to determine the risk of a fetus, maternal chromosomal deletions/duplications are allowed for the detection in practice. Moreover, the accuracy for screening fetal chromosomal deletions/duplications remained low, and investigation for the factors contributing to the high false positive rate is still warranted.

Among the true positive cases, seven cases occurred because of novel genetic changes, and other two cases were maternally inherited. In querying publicly available databases, eight true positive cases individually had a single pathogenic CNV, and another case was found to have a likely pathogenic 22q11.21 duplication. 22q11.21 duplication is a recurrent CNV with variable expressivity and incomplete penetrance (Olsen et al., 2018; Hoeffding et al., 2017). In the fetus with a confirmed 22q11.21 duplication, the CNV was inherited from the mother who exhibited no symptom up to now. This pregnant woman opted for continuing the pregnancy after posttest counseling. Moreover, another case is true positive on a pathogenic atypical 1 Mb 22q11.2 deletion, which was inherited from the mother with wide spectrum clinical features of 22q11.2 deletion syndrome. Other seven cases caught de novo pathogenic CNVs, and the pregnant women ultimately chose to terminate the pregnancy. Notably, identification of maternal CNVs is clinically relevant with the mother's own medical care. Also, heterozygous deletions/duplications of the parents have a 50% chance to be passed to the fetus. Thus, maternal chromosomal abnormality is an indication for invasive testing and a potential risk factor for future pregnancies. The increased risk of maternal chromosomal deletions/duplications might be considered if encountering the positive NIPT results for pathogenic CNVs.

In this work, we compared the performances of NIPT in detecting fetal chromosomal deletions/duplications at >10 Mb, between 5 and 10 Mb, and <5 Mb, respectively. The findings demonstrated that the PPV for the detection of CNVs < 5 Mb seemed to be higher than that of CNVs with larger size. However, several other studies demonstrated a better performance of NIPT for the detection of CNVs > 10 Mb (Hu et al., 2019; Yu et al., 2019). Furthermore, the reported CNVs in this study included recurrent and rare chromosomal deletions/duplications. Our results demonstrated that the PPV of recurrent CNVs seemed to be higher than that of rare chromosomal deletions/duplications. Moreover, the CNVs (<5 Mb) of two true positive cases (66.7%) were maternally inherited among this studied cohort. Notably, previous report has demonstrated that NIPT more easily detects maternal CNVs than that of fetus (Benn et al., 2015). In this study, a maternal 22q11.2 duplication contributed to the false NIPT positive result in one case. Thus, we speculated that maternal origin of chromosomal deletions/duplications was important factor affecting the accuracy in detecting fetal CNVs, especially for cases with CNVs smaller than 5 Mb.

Moreover, the follow‐up ultrasound examination identified fetal structural anomaly in two cases among positive pregnancies without invasive diagnosis. Though the etiologies of the fetal abnormality in these two cases were unclear, these results suggested that fetal ultrasound examination might benefit to the diagnosis of fetus with suspected chromosomal anomaly. Although cases with anatomic abnormality can be diagnosed on ultrasound identification, many cases might be missed or only identified relative late in pregnancy. Moreover, a 2q31.2 duplication (0.52 Mb) of uncertain significance was identified in a case with false positive 2q32.3q33.2 duplication. In another case with a false positive chromosomal duplication by NIPT, an unexpected pathogenic CNV was found by CMA. Thus, these results suggested that unexpected CNV might be found during invasive diagnostic testing, thereby increasing additional difficulties to genetic counseling. Another concern on the additional use of NIPT in chromosomal deletions/duplications was that the high false positive rate might lead to an increase of invasive testing.

## 5 CONCLUSION

In summary, the findings showed that the PPV of NIPT for chromosomal deletions/duplications detection in practice was low. When a positive NIPT result of clinically relevant chromosomal deletions/duplications was found via cffDNA sequencing, the potential maternal chromosomal abnormality might be considered. Addition to the specific chromosomal deletions/duplications, CMA might provide additional information on chromosomal anomalies of the fetus. For chromosomal deletions/duplications detection, genetic counseling is important, especially on the low accuracy of NIPT. Ultrasound examination can detect the structural anomalies caused by chromosomal aberrations, but was not able to identify the genetic etiologies and had limited ability to predict the future physical and intellectual development of the fetus.

## ETHICAL COMPLIANCE

This study was performed under the approval of the Ethics Committee of Xuzhou Maternity and Child Health Care Hospital Affiliated to Xuzhou Medical University.

## CONFLICT OF INTEREST

The authors report that they have no conflict of interest.

## AUTHOR CONTRIBUTIONS

L. Gou, F. Suo, and M. Gu designed the study, analyzed and interpreted the data, wrote, and revised the manuscript. Y. Wang, N. Wang, Q. Wu, S. Hu, P. Wang, L. Gu, M. Zhang, C. Wang, Y. Zhang, and X. Yin collected clinical samples and analyzed and interpreted the clinical data. P. Zhang, J. Xu, and X. Wang participated in the study design, data interpretation, and analysis, as well as the manuscript draft and revise. All authors reviewed the manuscript and approved the final version.

## Supporting information

Table S1

## Data Availability

The data that support the findings of this study are available on request from the corresponding author. The data are not publicly available due to privacy or ethical restrictions.
