# Supplementary material for: Clinical value for the detection of fetal chromosomal deletions/duplications by noninvasive prenatal testing in clinical practice
Source: Mol Genet Genomic Med. 2021 May 5;9(6):e1687. doi: 10.1002/mgg3.1687 (PMC8222853; doi:10.1002/mgg3.1687)
Supplement: Supplementary file 1 — Table S1 [file MGG3-9-e1687-s001.docx]

**Supplementary Table S1** NIPT results for chromosomal deletions/duplications without further validation by CMA or karyotpye analysis of amniocytes

| Sample  ID | MA  (Y) | GA  (W) | Clinical indications | NIPT results | CMA/karyotype analysis results | Maternal sequencing results | Classification  /follow up |
| --- | --- | --- | --- | --- | --- | --- | --- |
| Case 40 | 30 | 18 | Intermediate risk | Dup 4q25q28.1 (15.34 Mb) | ND | ND | NA |
| Case 41 | 34 | 19 | Intermediate risk | Del 5p15.33p15.2 (10.34 Mb) | ND | ND | NA |
| Case 42 | 34 | 18 | Intermediate risk | Dup 5p15.31 (2.08 Mb) | ND | ND | NA |
| Case 43 | 37 | 19 | NA | Dup 5p15.33 (1.84 Mb) | ND | ND | NA |
| Case 44 | 20 | 19 | Intermediate risk | Dup 5p15.33p15.1 (15.30 Mb); Dup 12q24.13q24.33 (18.47 Mb) | ND | ND | NA |
| Case 45 | 33 | 19 | Intermediate risk | Del 5q21.2q21.3 (5.13 Mb) | ND | ND | NA |
| Case 46 | 28 | 19 | Intermediate risk | Dup 8p23.2p23.3 (5.43 Mb) | ND | ND | NA |
| Case 47 | 19 | 21 | Intermediate risk | Del 9p22.1p22.3 (6.36 Mb) | ND | ND | NA |
| Case 48 | 39 | 18 | NA | Dup 11p12p11.12 (9.17 Mb) | ND | ND | NA |
| Case 49 | 30 | 16 | NA | Dup 12p13.33p11.1 (33.69 Mb) | ND | ND | Fetal structural abnormality; abortion |
| Case 50 | 30 | 20 | Intermediate risk | Dup 15q11.2q13.1 (4.82 Mb)  (15q11q13 recurrent region) | ND | Normal | NA |
| Case 51 | 23 | 21 | NA | Del 15q11.2q13.1 (5.69 Mb)  (15q11q13 recurrent region) | ND | ND | NA |
| Case 52 | 28 | 19 | Intermediate risk | Dup 16p11.2q12.2 (6.16 Mb) | ND | ND | NA |
| Case 53 | 43 | 18 | Advanced maternal age | Del 21q21.3q22.11 (3.85 Mb) | ND | ND | NA |
| Case 54 | 29 | 20 | NA | Dup 22q11.22q11.23 (0.72 Mb)  (22q11.2 recurrent region) | ND | ND | NA |
| Case 55 | 36 | 17 | Advanced maternal age | Dup 22q11.21 (1.42 Mb)  (22q11.2 recurrent region) | ND | ND | NA |
| Case 56 | 29 | 17 | Intermediate risk | Dup 22q11.2 (4.71 Mb)  (22q11.2 recurrent region) | ND | ND | NA |
| Case 57 | 29 | 17 | Intermediate risk | Dup 22q11.21q11.3 (4.71 Mb)  (22q11.2 recurrent region) | ND | ND | NA |
| Case 58 | 30 | 21 | Intermediate risk | Dup 22q11.21q12.1 (13.10 Mb)  (22q11.2 recurrent region) | ND | ND | Fetal structural abnormality; abortion |
| Case 59 | 25 | 19 | Intermediate risk | Del 22q11.21 (1.52 Mb)  (22q11.2 recurrent region) | ND | ND | NA |
| Case 60 | 34 | 18 | High risk | Del 22q11.22q11.23 (0.72 Mb)  (22q11.2 recurrent region) | ND | ND | NA |

CMA, chromosomal microarray analysis; CNVs, copy number variants; Del, deletion; Dup, duplication; GA, gestational age; MA, maternal age; NA, not available; ND, not detected; NIPT, noninvasive prenatal testing; W, week; Y, year.
